# Supplementary material for: Predicting The Pathway Involvement Of Metabolites Based on Combined Metabolite and Pathway Features
Source: bioRxiv. 2024 Apr 2:2024.04.01.587582. Preprint. [Version 1] doi: 10.1101/2024.04.01.587582 (PMC11014601; doi:10.1101/2024.04.01.587582)
Supplement: Supplement 1 [file NIHPP2024.04.01.587582v1-supplement-1.pdf]

Table S1 – Hyperparameters.

Model	Encoded	Hyperparameter Name	Hyperparameter Search Range	Hyperparameter Value Selected
XGBoost	True	Subsample	[0.7, 1.0]	0.964
		Max depth	[15, 25]	17
		ETA	[0.3, 0.5]	0.317
		Minimum split loss	[0.0, 0.8]	0.0159
		Alpha	[1.7, 3.0]	1.94
		Lambda	[1.9, 2.5]	2.46
	False	Subsample	[0.7, 1.0]	0.983
		Max depth	[15, 25]	18
		ETA	[0.3, 0.5]	0.319
		Minimum split loss	[0.0, 0.8]	0.000533
		Alpha	[1.7, 3.0]	1.72
		Lambda	[1.9, 2.5]	2.46
MLP	True	Activation function	[SELU, CELU, ELU, PReLU, Leaky ReLU, RReLU, ReLU, ReLU6, SiLU]	PReLU
		Normalization layer type	[Batch Normalization, Layer Normalization, Instance Normalization, None]	Batch Normalization
		Loss function	[Huber, Binary Cross Entropy]	Binary Cross Entropy
		Hidden dimension size	[50, 4000]	479
		Number of layers	[1, 6]	6
		Dropout	[0.0, 0.1]	0.0498
		Learning rate	[10^-6, 10^-3]	0.000245
		Beta 1	[0.0, 0.9999]	0.963
		Beta 2	[0.0, 0.9999]	0.660
		Epsilon	[10^-10, 10^-3]	0.000769
		Weight decay	[0.0, 0.2]	2.89 x 10^-5
		Classification threshold	[0.3, 0.7]	0.671
	False	Activation function	[SELU, CELU, ELU, PReLU, Leaky ReLU, RReLU, ReLU, ReLU6, SiLU]	PReLU
		Normalization layer type	[Batch Normalization, Layer Normalization, Instance Normalization, None]	Batch Normalization
		Loss function	[Huber, Binary Cross	Huber

			Entropy]	
		Hidden dimension size	[50, 4000]	706
		Number of layers	[1, 6]	5
		Dropout	[0.0, 0.1]	0.0691
		Learning rate	[10^-6, 10^-3]	0.000273
		Beta 1	[0.0, 0.9999]	0.633
		Beta 2	[0.0, 0.9999]	0.616
		Epsilon	[10^-10, 10^-3]	3.95x10^-6
		Weight decay	[0.0, 0.2]	4.30x10^-5
		Classification threshold	[0.3, 0.7]	0.493

Figure S1 – Feature importance scores of pathway features compared to corresponding metabolite features.

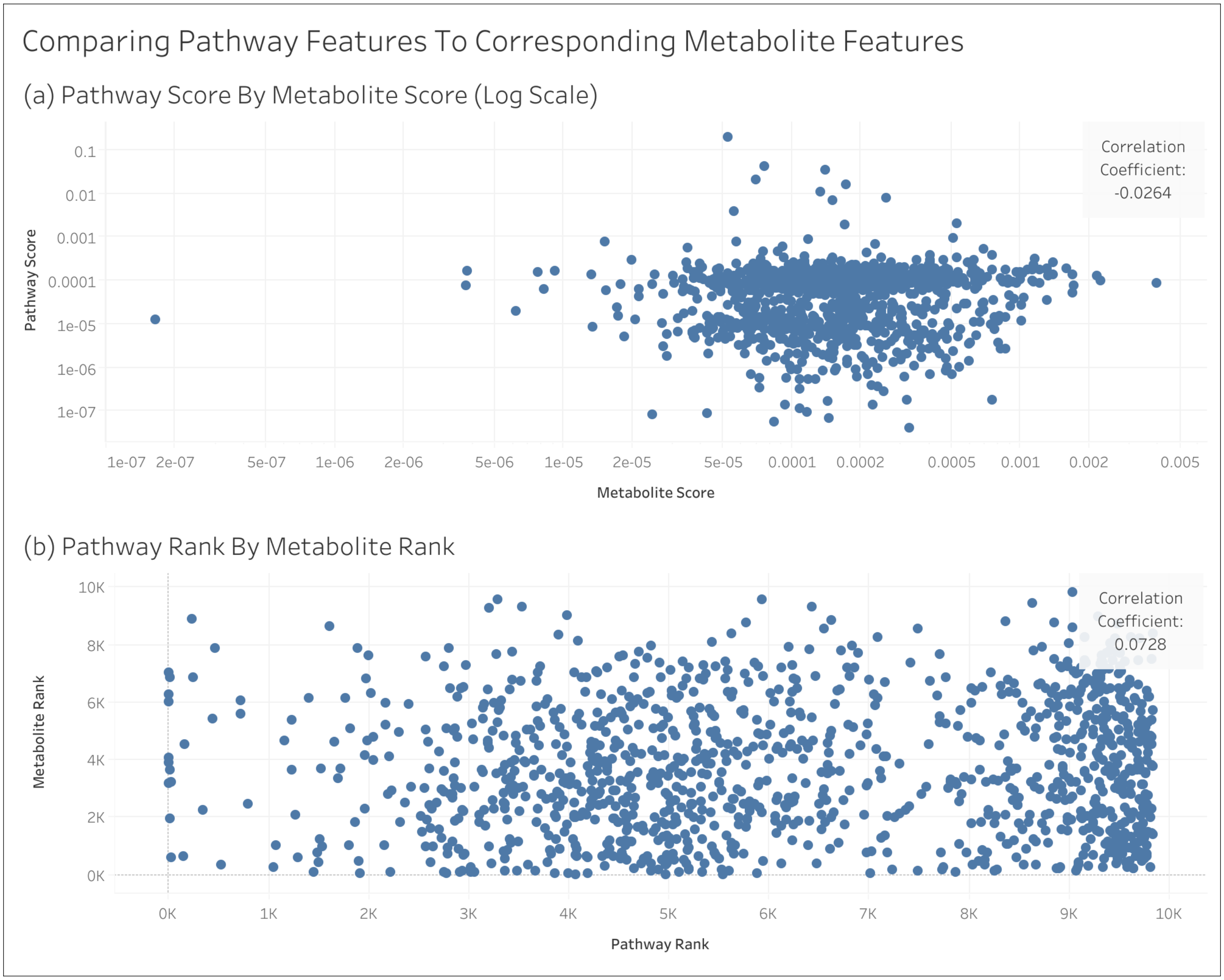

Table S2 - All Metrics And Scores.

Model	Encoded	Pathway Category	Metric	Average Score	Standard Deviation

MLP	False	ALL	Accuracy	0.9592	0.0027
			F1 score	0.8069	0.0114
			MCC	0.7844	0.0129
			Precision	0.8128	0.0224
			Recall	0.8019	0.0193
		Amino acid metabolism	Accuracy	0.9380	0.0103
			F1 score	0.7027	0.0485
			MCC	0.6712	0.0518
			Precision	0.7250	0.0711
			Recall	0.6904	0.0761
		Biosynthesis of other secondary metabolites	Accuracy	0.9329	0.0107
			F1 score	0.8716	0.0202
			MCC	0.8268	0.0268
			Precision	0.8710	0.0354
			Recall	0.8737	0.0305
		Carbohydrate metabolism	Accuracy	0.9619	0.0089
			F1 score	0.7920	0.0473
			MCC	0.7736	0.0504
			Precision	0.7849	0.0716
			Recall	0.8074	0.0732
		Chemical structure transformation maps	Accuracy	0.9149	0.0126
			F1 score	0.4295	0.0667
			MCC	0.3879	0.0696
			Precision	0.4539	0.0838
			Recall	0.4193	0.0868
		Energy metabolism	Accuracy	0.9754	0.0067
			F1 score	0.5751	0.1031
			MCC	0.5707	0.1035
			Precision	0.6152	0.1400
			Recall	0.5662	0.1351
		Glycan biosynthesis and metabolism	Accuracy	0.9729	0.0073
			F1 score	0.7674	0.0568
			MCC	0.7560	0.0589
			Precision	0.7562	0.0858
			Recall	0.7894	0.0801
		Lipid metabolism	Accuracy	0.9792	0.0074

			F1 score	0.9138	0.0288
			MCC	0.9029	0.0320
			Precision	0.9094	0.0507
			Recall	0.9210	0.0373
		Metabolism of cofactors and vitamins	Accuracy	0.9555	0.0087
			F1 score	0.7620	0.0451
			MCC	0.7403	0.0481
			Precision	0.7897	0.0712
			Recall	0.7435	0.0691
		Metabolism of other amino acids	Accuracy	0.9650	0.0079
			F1 score	0.6042	0.0802
			MCC	0.5937	0.0813
			Precision	0.6738	0.1159
			Recall	0.5631	0.1021
		Metabolism of terpenoids and polyketides	Accuracy	0.9646	0.0081
			F1 score	0.9080	0.0206
			MCC	0.8867	0.0251
			Precision	0.9087	0.0363
			Recall	0.9089	0.0316
		Nucleotide metabolism	Accuracy	0.9866	0.0051
			F1 score	0.7685	0.0835
			MCC	0.7680	0.0816
			Precision	0.7934	0.1259
			Recall	0.7680	0.1187
		Xenobiotics biodegradation and metabolism	Accuracy	0.9634	0.0089
			F1 score	0.8884	0.0262
			MCC	0.8677	0.0305
			Precision	0.8947	0.0470
			Recall	0.8853	0.0431
	True	ALL	Accuracy	0.9551	0.0032
			F1 score	0.7942	0.0124
			MCC	0.7695	0.0139
			Precision	0.7759	0.0235
			Recall	0.8140	0.0177
		Amino acid metabolism	Accuracy	0.9240	0.0140
			F1 score	0.6699	0.0486
			MCC	0.6327	0.0520

			Precision	0.6344	0.0773
			Recall	0.7228	0.0790
		Biosynthesis of other secondary metabolites	Accuracy	0.9265	0.0123
			F1 score	0.8597	0.0224
			MCC	0.8110	0.0296
			Precision	0.8577	0.0422
			Recall	0.8641	0.0344
		Carbohydrate metabolism	Accuracy	0.9592	0.0095
			F1 score	0.7879	0.0444
			MCC	0.7694	0.0467
			Precision	0.7487	0.0724
			Recall	0.8400	0.0640
		Chemical structure transformation maps	Accuracy	0.9199	0.0178
			F1 score	0.5139	0.0601
			MCC	0.4789	0.0621
			Precision	0.5049	0.0956
			Recall	0.5505	0.1069
		Energy metabolism	Accuracy	0.9755	0.0065
			F1 score	0.5878	0.0973
			MCC	0.5814	0.0982
			Precision	0.6065	0.1288
			Recall	0.5924	0.1253
		Glycan biosynthesis and metabolism	Accuracy	0.9740	0.0065
			F1 score	0.7763	0.0546
			MCC	0.7650	0.0565
			Precision	0.7635	0.0793
			Recall	0.7979	0.0749
		Lipid metabolism	Accuracy	0.9770	0.0066
			F1 score	0.9053	0.0263
			MCC	0.8930	0.0292
			Precision	0.8943	0.0450
			Recall	0.9188	0.0358
		Metabolism of cofactors and vitamins	Accuracy	0.9477	0.0100
			F1 score	0.7264	0.0481
			MCC	0.7001	0.0517
			Precision	0.7387	0.0751

			Recall	0.7228	0.0701
		Metabolism of other amino acids	Accuracy	0.9590	0.0095
			F1 score	0.5890	0.0796
			MCC	0.5733	0.0812
			Precision	0.5819	0.1083
			Recall	0.6168	0.1104
		Metabolism of terpenoids and polyketides	Accuracy	0.9605	0.0089
			F1 score	0.8979	0.0227
			MCC	0.8741	0.0275
			Precision	0.8930	0.0399
			Recall	0.9047	0.0334
		Nucleotide metabolism	Accuracy	0.9853	0.0059
			F1 score	0.7584	0.0866
			MCC	0.7571	0.0856
			Precision	0.7513	0.1305
			Recall	0.7889	0.1126
		Xenobiotics biodegradation and metabolism	Accuracy	0.9526	0.0112
			F1 score	0.8604	0.0299
			MCC	0.8339	0.0347
			Precision	0.8410	0.0569
			Recall	0.8853	0.0452
XGBoost	False	ALL	Accuracy	0.9564	0.0023
			F1 score	0.7871	0.0115
			MCC	0.7637	0.0126
			Precision	0.8185	0.0134
			Recall	0.7583	0.0159
		Amino acid metabolism	Accuracy	0.9267	0.0105
			F1 score	0.6506	0.0484
			MCC	0.6110	0.0525
			Precision	0.6640	0.0584
			Recall	0.6415	0.0616
		Biosynthesis of other secondary metabolites	Accuracy	0.9156	0.0113
			F1 score	0.8368	0.0224
			MCC	0.7803	0.0293
			Precision	0.8437	0.0308
			Recall	0.8311	0.0300
		Carbohydrate metabolism	Accurac	0.9641	0.0077

			y		
			F1 score	0.8042	0.0421
			MCC	0.7857	0.0454
			Precisio n	0.7911	0.0570
			Recall	0.8214	0.0539
		Chemical structure transformation maps	Accurac y	0.9289	0.0104
			F1 score	0.4462	0.0692
			MCC	0.4221	0.0722
			Precisio n	0.5645	0.0933
			Recall	0.3743	0.0711
		Energy metabolism	Accurac y	0.9776	0.0061
			F1 score	0.5532	0.1105
			MCC	0.5578	0.1090
			Precisio n	0.6962	0.1370
			Recall	0.4723	0.1220
		Glycan biosynthesis and metabolism	Accurac y	0.9763	0.0062
			F1 score	0.7800	0.0581
			MCC	0.7701	0.0599
			Precisio n	0.8219	0.0699
			Recall	0.7476	0.0790
		Lipid metabolism	Accurac y	0.9780	0.0058
			F1 score	0.9056	0.0248
			MCC	0.8938	0.0276
			Precisio n	0.9270	0.0314
			Recall	0.8864	0.0378
		Metabolism of cofactors and vitamins	Accurac y	0.9540	0.0082
			F1 score	0.7336	0.0474
			MCC	0.7157	0.0491
			Precisio n	0.8298	0.0560
			Recall	0.6611	0.0636
		Metabolism of other amino acids	Accurac y	0.9589	0.0080
			F1 score	0.5181	0.0835
			MCC	0.5042	0.0856
			Precisio n	0.5974	0.1066
			Recall	0.4660	0.0920
		Metabolism of terpenoids and polyketides	Accurac y	0.9579	0.0082
			F1 score	0.8881	0.0226

			MCC	0.8628	0.0271
			Precision	0.9060	0.0278
			Recall	0.8719	0.0329
		Nucleotide metabolism	Accuracy	0.9874	0.0045
			F1 score	0.7754	0.0807
			MCC	0.7729	0.0807
			Precision	0.8128	0.1003
			Recall	0.7531	0.1086
		Xenobiotics biodegradation and metabolism	Accuracy	0.9516	0.0084
			F1 score	0.8510	0.0266
			MCC	0.8226	0.0309
			Precision	0.8615	0.0346
			Recall	0.8421	0.0371
	True	ALL	Accuracy	0.9414	0.0023
			F1 score	0.6747	0.0149
			MCC	0.6567	0.0152
			Precision	0.8233	0.0166
			Recall	0.5718	0.0184
		Amino acid metabolism	Accuracy	0.9106	0.0116
			F1 score	0.4507	0.0611
			MCC	0.4342	0.0628
			Precision	0.6598	0.0845
			Recall	0.3455	0.0584
		Biosynthesis of other secondary metabolites	Accuracy	0.8850	0.0128
			F1 score	0.7591	0.0281
			MCC	0.6897	0.0344
			Precision	0.8355	0.0337
			Recall	0.6966	0.0369
		Carbohydrate metabolism	Accuracy	0.9501	0.0087
			F1 score	0.6930	0.0521
			MCC	0.6723	0.0542
			Precision	0.7792	0.0637
			Recall	0.6283	0.0674
		Chemical structure transformation maps	Accuracy	0.9253	0.0108
			F1 score	0.2238	0.0729
			MCC	0.2580	0.0803
			Precisio	0.5831	0.1563

			Precision		
			Recall	0.1413	0.0518
		Energy metabolism	Accuracy	0.9747	0.0064
			F1 score	0.3916	0.1231
			MCC	0.4312	0.1211
			Precision	0.7222	0.1808
			Recall	0.2781	0.1042
		Glycan biosynthesis and metabolism	Accuracy	0.9715	0.0068
			F1 score	0.7056	0.0663
			MCC	0.7040	0.0653
			Precision	0.8522	0.0736
			Recall	0.6072	0.0816
		Lipid metabolism	Accuracy	0.9612	0.0080
			F1 score	0.8184	0.0376
			MCC	0.8045	0.0387
			Precision	0.9272	0.0344
			Recall	0.7344	0.0535
		Metabolism of cofactors and vitamins	Accuracy	0.9344	0.0094
			F1 score	0.5415	0.0624
			MCC	0.5504	0.0594
			Precision	0.8287	0.0741
			Recall	0.4054	0.0623
		Metabolism of other amino acids	Accuracy	0.9556	0.0084
			F1 score	0.3361	0.0965
			MCC	0.3589	0.0990
			Precision	0.6100	0.1556
			Recall	0.2380	0.0801
		Metabolism of terpenoids and polyketides	Accuracy	0.9320	0.0101
			F1 score	0.8084	0.0298
			MCC	0.7717	0.0338
			Precision	0.8808	0.0333
			Recall	0.7484	0.0422
		Nucleotide metabolism	Accuracy	0.9785	0.0059
			F1 score	0.5307	0.1220
			MCC	0.5465	0.1184
			Precision	0.7403	0.1546
			Recall	0.4263	0.1234

		Xenobiotics biodegradation and metabolism	Accuracy	0.9182	0.0113
			F1 score	0.7177	0.0398
			MCC	0.6800	0.0428
			Precision	0.8294	0.0438
			Recall	0.6348	0.0524
